# HIF-1-Induced hsa-miR-429: Understanding Its Direct Targets as the Key to Developing Cancer Diagnostics and Therapies

**DOI:** 10.3390/cancers15112903

**Published:** 2023-05-25

**Authors:** Sylwia Bartoszewska, Jakub Sławski, James F. Collawn, Rafal Bartoszewski

**Affiliations:** 1 Department of Inorganic Chemistry, Medical University of Gdansk, 80-416 Gdansk, Poland; 2 Department of Biophysics, Faculty of Biotechnology, University of Wroclaw, 50-383 Wroclaw, Poland; 3 Department of Cell, Developmental and Integrative Biology, University of Alabama, Birmingham, AL 35294, USA

**Keywords:** ncRNAs, microRNAs, siRNAs, RNAi therapy, RNAi drug candidates, off-target effects

## Abstract

**Simple Summary:**

In this review, we discuss how miRNAs play a critical role in the regulation of mRNA stability and translation, how determining the direct targets of miRNAs in complex networks is extremely difficult, and how translating specific miRNAs to the clinic often results in failure. This has led to concerns that this approach may not be feasible. Using hsa-miR-429 as an example, we discuss the limitations encountered in the development of efficient miRNAs-related therapies and diagnostic approaches and how this can be improved. We also provide a literature analysis of the verified hsa-miR-429 targets in various human research models. A meta-analysis of this work should provide better insights into the role of hsa-miR-429 in cancer diagnosis and any potential therapeutic approaches.

**Abstract:**

MicroRNAs (miRNAs) play a critical role in the regulation of mRNA stability and translation. In spite of our present knowledge on the mechanisms of mRNA regulation by miRNAs, the utilization and translation of these ncRNAs into clinical applications have been problematic. Using hsa-miR-429 as an example, we discuss the limitations encountered in the development of efficient miRNA-related therapies and diagnostic approaches. The miR-200 family members, which include hsa-miR-429, have been shown to be dysregulated in different types of cancer. Although these miR-200 family members have been shown to function in suppressing epithelial-to-mesenchymal transition, tumor metastasis, and chemoresistance, the experimental results have often been contradictory. These complications involve not only the complex networks involving these noncoding RNAs, but also the problem of identifying false positives. To overcome these limitations, a more comprehensive research strategy is needed to increase our understanding of the mechanisms underlying their biological role in mRNA regulation. Here, we provide a literature analysis of the verified hsa-miR-429 targets in various human research models. A meta-analysis of this work is presented to provide better insights into the role of hsa-miR-429 in cancer diagnosis and any potential therapeutic approach.

## 1. Introduction

Short, endogenous non-coding double-stranded microRNAs (miRNAs) are involved in the repressive control of mRNA translation [1,2,3,4,5]. Understanding their mechanism of action in modulating mRNA stability and translation has opened new prospects for the diagnosis and therapy of human diseases, including cancers. Notably, the modulation of miRNA levels not only allows for the elimination of dysregulated proteins but also provides the opportunity to restore physiological proteostasis [6,7,8,9,10,11,12,13,14,15,16,17,18,19,20,21,22,23,24]. Despite studies continuously reporting novel examples of disease-related dysregulation of miRNAs levels and novel targets of these molecules, the diagnostic approaches and clinical trials have often been unsuccessful [6,7,25,26,27,28,29,30,31]. These studies have led to skepticism regarding any possible miRNA clinical translational possibilities. 

This specificity problem could result from a number of reasons including the following: (1) mammalian miRNAs are not required to be perfectly complementary to their target mRNA sequences; (2) each miRNA can affect up to hundreds of targets; (3) miRNAs can promote indirect effects through the modulation of transcription factors (TFs) and the inhibition of their downstream targets, thus generating false positives [32,33,34]. To make matters worse, mRNA stability and translational efficiency can be modulated by different miRNAs simultaneously. Furthermore, although some miRNAs have been shown to be potent post-transcriptional repressors [35,36,37,38,39,40,41,42,43,44,45,46,47,48], the vast majority of these molecules only provide a modest modulation of the subsequent protein levels and thus serve more as ‘molecular buffers’ than as strong repressors of gene expression [49,50]. Therefore, understanding the complex molecular networks modulated by miRNAs remains a challenging, but mandatory, step towards any successful utilization of these molecules in any therapeutic or diagnostic approach. 

**Figure 1:**
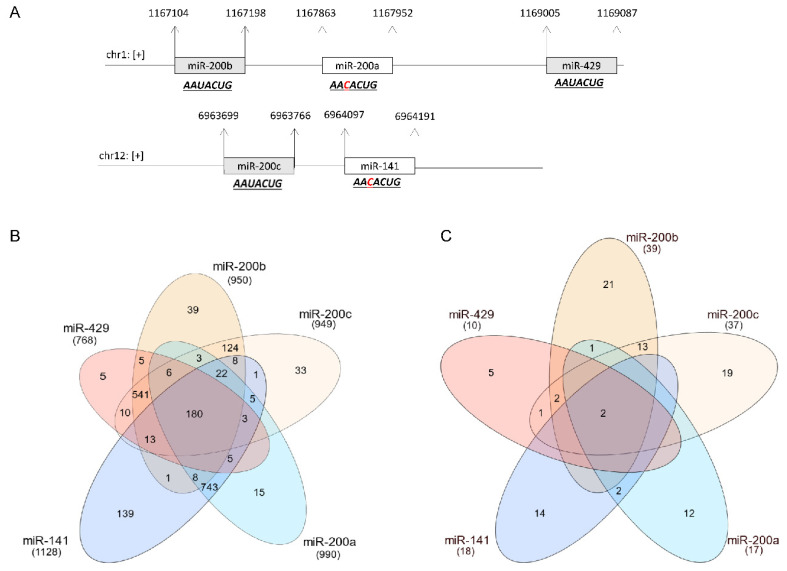
Genetic organization of the miR-200 family in humans. (**A**) The chromosome loci of the two clusters of the miR-200 family with their seed sequences are presented in boxes. The seed sequences differ by one nucleotide (indicated in red). (**B**) Venn diagram representing the distribution overlap of the top 1% of predicted targets (*p*-value < 0.01, mirDIP webserver) of the miR-200 family members. (**C**) Venn diagram representing the distribution overlap of experimentally confirmed targets (mirTarbase webserver) of the miR-200 family members.

In this review, we discuss the molecular networks of miR-429, a member of the miR-200 family that is induced by HIF-1 during hypoxia [51]. miR-429 is commonly associated with the progression and metastatic potential of many human cancers [52,53,54,55,56,57]. The members of the miR-200 family are clustered in two genomic locations: one on chromosome 1 (1p36.33) that comprises miR-200b, miR-200a and miR-429, and the other on chromosome 12 (12p13.31) that includes miR-200c and miR-141 (Figure 1A) [58]. Despite the fact that these locations encode harpin precursors for both 5′ and 3′ miR-200 family members, the 3’ mature miRNA forms are the dominant ones [58], and therefore the -3p postfix is omitted in the majority of reports including in this review. 

Although the miR-200 family members have been widely accepted as tumor suppressors, these studies have been controversial with regard to cancer prognoses [52,56,57]. Here, we discuss hsa-miR-429 and the challenges in understanding this miRNA role in cancer development. The first problem is the similarity of seed sequences in all of the miR-200 family members. This makes determining the specificity of each of the different members of this family a challenge. Certainly, not properly addressing this in an experimental model could eventually lead to false conclusions. The seed sequence AAUACUG is common for miRs-200b, miR-200c and miR-429, and there is only a one-nucleotide sequence difference, AACACUG, in miR-200a and miR-141 [23,58]. This identity and similarity make the mRNA target profiles of this family of miRNAs very similar (Figure 1).

As shown in Figure 1B, the vast majority of the predicted miR-429 high-probability targets (top 5%) are common for all of the miR-429 family members. The only difference is the “unique targets” of miR-429 can also be recognized with a probability higher than 95% [59,60]. A similar picture was obtained for miR-429 targets that were verified experimentally (Figure 1C) [61]. Furthermore, the expression of specific miR-200 family members such as hsa-miR-429 is modulated by the cellular context, cellular stress conditions, and the stage of tumor progression and metastasis [52,53,54,55,56,57]. How this relates to the other family members is still unclear. 

Although the miR-200 family members including hsa-miR-429 were shown in numerous reports to be dysregulated in different types of cancer and assigned functions in suppressing epithelial–mesenchymal transition (EMT), tumor metastasis and chemoresistance [52,53,54,55,56,57], the data are often contradictory and difficult to understand. Therefore, a systematic analysis of the molecular network of their targets and context is needed. Here, we provide a comprehensive literature and meta-analysis of the reports verifying hsa-miR-429 targets in various human research models. 

## 2. Methods

### 2.1. Literature Search Strategy

The review was prepared according to recommendations for systematic reviews and meta-analyses [62,63]. This comprehensive literature search included two online databases, PubMed and Scopus, up to February 2023. The terms “miR-429, hsa-miR-429, microRNA-429, miR-429-3p, hsa-miR-429-3p” were used as search identifiers in the relevant literature search. 

### 2.2. Inclusion and Exclusion Criteria

**Figure 2:**
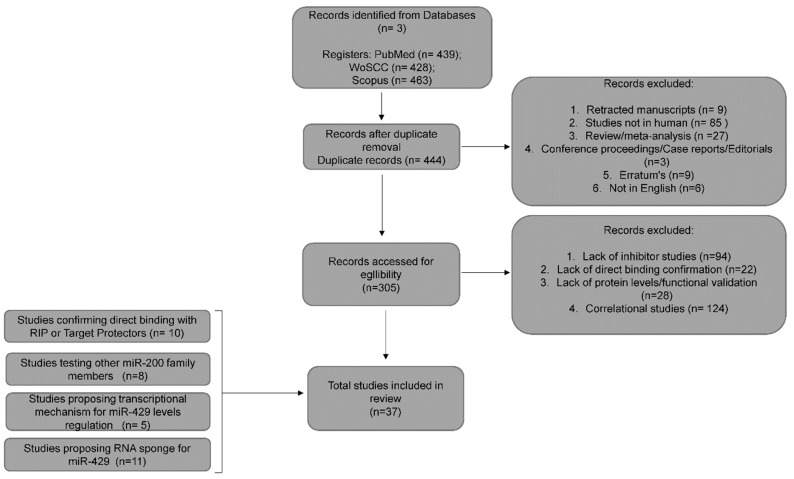
Flowchart of the literature search and identification of targets discussed in this study.

The studies identified in these searches were included for further evaluation when the following criteria were fulfilled: (1) miR-429 was investigated in any type of human cell type; (2) the experimental approach aimed to determine miR-429 direct mRNA targets; (3) the study included experimental confirmation of miR-429 direct binding to the target sequence using a target mask (protector), luciferase reporters or RNA immunoprecipitation; (4) the target validation included miR-429 overexpression by synthetic analogs, such as mimics, pre-miRs, or vectors and inhibition by specific miR-429 inhibitors. Furthermore, studies were excluded according to following criteria: (1) non-English articles; (2) retracted articles; (3) errata and other types of articles such as conference records, abstracts, reviews or metanalyses; (4) insufficient data to confirm direct miR-429 interactions; (5) the prediction data were obtained from TCGA and GEO datasets. The schematic workflow of the data selection is presented in Figure 2.

### 2.3. Data Extraction

All included studies were independently identified by two investigators (S.B. and R.B.), and uncertain data were verified by the third author (J.C.F). The following information was collected: (1) authors names, (2) DOI, (3) publication year, (4) sample types, (5) cell types, (6) type of miRNA, (7) type of biochemical strategy used to verify hsa-miR-429 targets, (8) assays used to evaluate functional role of identified interactions and (9) translational models. 

### 2.4. Prediction of mRNA Targets and Gene Ontology Analysis

The miRNA–mRNA target interactions were predicted and analyzed with the use of mirDIP (microRNA Data Integration Portal and miRTarBase). The mirDIP database provides nearly 152 million human miRNA–target predictions, which were collected across 30 different resources and does not accumulate prediction bias toward biological processes or pathways [60,61]. miRTarBase contains miRNA–target interactions which were collected by manually surveying pertinent literature [61]. The Enrichr (https://amp.pharm.mssm.edu/Enrichr/, accessed on 2 February 2023) [64] and WebGestalt (https://www.webgestalt.org/, accessed on 2 February 2023) [65] webservers were applied to the analysis results into the “Gene Ontology categories” with the selection based on a *Q* value < 0.05. Furthermore, the analyses were limited to experimentally verified interactions, and no extended gene enrichment set analyses were performed. 

## 3. Experimental Approaches to Define the Molecular Network of hsa-miR-429

The identification and solid experimental verification of both the mRNA targets as well as the transcriptional and posttranscriptional regulators of a specific miRNAs remain a major obstacle to understand their role in cellular signaling pathways. These types of analyses are mandatory for the further efficient translation of miRNAs into therapeutic approaches. Hence, although many reports regarding the mRNA targets and the biological function of hsa-miR-429 accumulated over the last decade, here we focused only on the rigorous ones that were based on experimental verification. 

There is no single experimental method, unfortunately, that provides researchers with total confidence that any miRNA binding to mRNA is physiologically relevant. Therefore, multiple independent experimental approaches are required. For example, miRNA overexpression with the use of synthetic analogs (miRNA mimics containing the sequences that are identical to the guide strands of the mature miRNAs) results in the dramatic and non-physiological overexpression (from hundred- to thousandfold) of these molecules in cells. This level of overexpression oversaturates the RNA-induced silencing complexes (RISC complexes) and results in binding to mRNA targets that may not be physiologically relevant [66,67]. Alternative strategies to limit the miRNA overexpression effects on target gene expression in a more physiologically appropriate way include designing longer synthetic miRNA precursors such as pre-miRNAs that undergo Dicer processing in the cytoplasm or the use of pri-miRNAs that require delivery to the nucleus for processing [68,69,70,71].

A similar false positive bias can be generated by luciferase reporter approaches in which easily transfected human cell lines such as HEK293 cells serve as an environment for vectors expressing target 3’UTRs attached to the luciferase reporter as well as the miRNA analogs being tested [67,72,73,74,75,76]. In this type of system, both the target sequences and the miRNAs are elevated to nonphysiological levels, and the cell serves as the source of the RNAi machinery. Thus, even if mutagenesis, which often relies on removing the entire target sequence, confirms a potential miRNA–mRNA binding, it still needs to be considered as an overexpression approach. 

Another approach for monitoring miRNA–mRNA interactions can be achieved by the utilization of specific miRNA inhibitors that impair the endogenous miRNA levels. AntagomiRs are synthetic chemically modified ssRNAs that are fully complementary miRNAs and thereby effectively sequester mature miRNAs [77,78]. The mechanism of miRNA inhibition by these molecules depends on the type of chemical modifications used. For example, high-affinity inhibitors form heteroduplexes with the targeted miRNAs, whereas low-affinity ones promote miRNA degradation [79,80,81,82]. There are concerns even about the use of antagomiRs. For example, this approach affects the entire miRNA target network, and this could allow other genes to modulate the expression of the candidate target and therefore lead to false positives based on indirect effects [83]. 

A more specific and appropriate approach to verify mRNA targets utilizes target protectors (morpholinos) that prevent endogenous miRNA binding to an mRNA region containing a specific miR seed sequence [84]. These molecules are ssRNAs (of ~25 bases), chemically modified to prevent them from triggering the RNAi pathway, and are complementary to an mRNA target sequence (of at least 14–15 contiguous bases). This approach blocks the relevant mRNA in question and provides the needed specificity that the other approaches lack [8,84,85,86,87]. That being said, although they provide a better physiological insight into miRNA–mRNA interactions, it remains possible that the binding in the 3′ mRNA regions may contain other miRNA target sites that could still be affected. If the target protector blocks the significant decreases in the target mRNA expression, then this concern is minimized. This target protector approach, unfortunately, would not differentiate between the targets of miR-429 and other miR-200 family members. 

Another viable approach to verify miRNA–mRNA interactions relies on analyzing the composition of RISC complexes (RNA Immunoprecipitation Chip Assay, RIP assay) [88,89]. In these assays, immunoprecipitation of Argonaute RISC Catalytic Component 2 (Ago2), containing the other RISC components including all of the complexed mRNAs and miRNAs, is carried out. The verification of the miRNA and mRNA of interest is verified by qPCR or Next-Generation Sequencing (NGS) [50]. Although these assays are a solid prognostic of functional miRNA–mRNA interactions, the RISC complexes reflect all of the bound miRNAs and mRNAs in the cells. Therefore, the limitation of this approach is that, though it identifies the mRNA and miRNA contained in the RISC, it does not demonstrate that the two components actually exist in a functional hybrid. Furthermore, numerous research applications ignore the fact that even if the miRNA-related mRNA expression levels do change, it does not mean that this will lead to a change in the protein levels or the phenotype [90]. 

**Figure 3:**
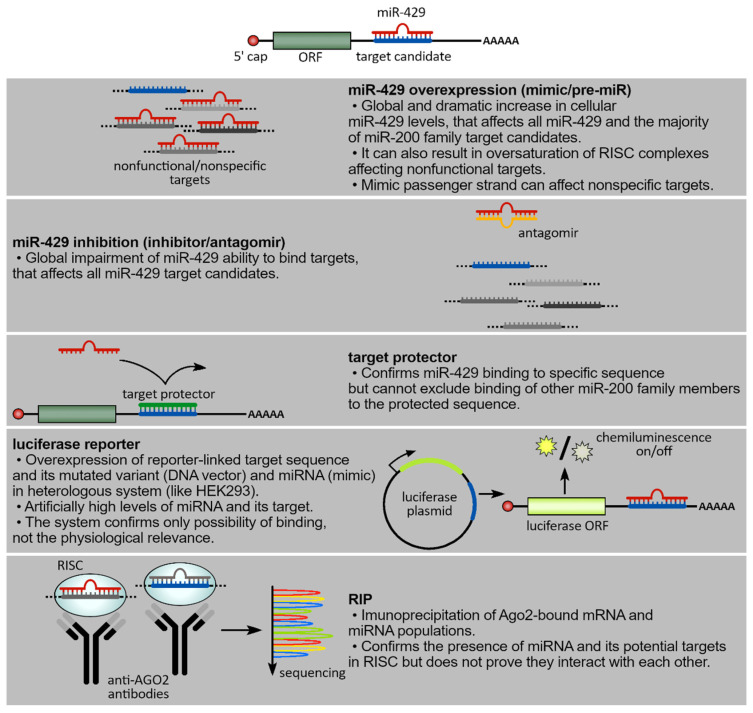
Schematic comparison of different experimental approaches to verify miRNA–mRNA target direct binding.

Taken together, the verification of miRNA–mRNA interactions is a complex challenge that should be based on multiple independent experimental strategies (Figure 3). One should also consider process-related changes in the endogenous miRNA levels as well as the complexity of signaling pathways that the miRNA’s target mRNA plays. Despite the large number of reports regarding hsa-miR-429 function, our inclusion criteria resulted in a final selection of 47 genes (Figure 2) that were identified either as a direct target of hsa-miR-429 or as transcriptional or posttranscriptional modulators, e.g., transcription factors or lncRNAs (sponges), respectively (Table 1).

To obtain insights into the biological role of the hsa-miR-429 network, we performed gene enrichment analyses using two well established integrative web tools, Enrichr and Webgestalt [64,65], and applied a strict False Discovery Rate-based (FDR) criteria analysis [133,134]. This approach assigned the hsa-miR-429-related target genes and indicated that this miRNA is an important regulator of processes that are either activated during development or dysregulated in human cancer and cardiovascular diseases.

## 4. hsa-miR-429 and Cancer

The ability of epithelial cells to reversibly alter the expression of their cell adhesion proteins and their cytoskeleton to become motile mesenchymal cells is manifested during EMT. EMT is an essential and strictly regulated process in normal embryonic development, that includes the establishment of the neural crest and gastrulation, as well as tissue regeneration in adults [135]. Cancer cells, however, utilize an aberrant, partial reactivation of EMT in order to allow for the invasion of primary tumor cells [135,136,137]. Notably, hsa-miR-429 along with the other miR-200 family members have been identified as important modulators of the EMT through their interaction with the zinc finger E-box binding homeobox proteins ZEB1 and ZEB2 [53,56,91,92,93,96]. Furthermore, follow-up studies extended the regulatory role of hsa-miR-429 in this pathway to direct network interactions with AKT serine/threonine kinase 1 (*AKT1*) [108], the enhancer of zeste 2 polycomb repressive complex 2 subunit (*EZH2*) [123], the notch receptor 1 (*NOTCH1*) [111], occludin (*OCLN*) [106], RB binding protein 4, chromatin remodeling factor (*RBBP4*) [132], tight junction protein 1 (*TJP1*) [106] and hypoxia inducible factor 1 subunit alpha (*HIF1A*) [101,102,103]. 

In many cancers models, the repression of the endogenous levels of hsa-miR-429 leads to increased levels of ZEB1 and ZEB2 and consequently to reduced E-cadherin synthesis and increased cellular motility [138]. This mechanism is even more interesting in light of reports showing that all miR-200 family members are directly repressed transcriptional targets during ZEB1 and ZEB2 activation [139,140]. Thus, hsa-miR-429, along with the others miR-200 family members, and ZEB1 or ZEB2 function within a double-negative feedback loop, and consequently, changes in the levels of the miRNA or the ZEB factors have dramatic effects on each other. Thus, depending on the cellular signaling network, a cell can switch to either the epithelial or the mesenchymal phenotype [138]. 

The cellular signals that initiate increased ZEB factor expression and the related feedback loop activation include the TGF-β and TNF-α signaling pathways and the cellular responses from steroid hormones and hypoxia [138]. Interestingly, hsa-miR-429 participates in a negative feedback loop as an important direct regulator of HIF-1 signaling in normal human endothelial cells [101,103,141]. Thus, hsa-miR-429 could work as an additional safety mechanism by preventing extensive HIF-1-driven expression of ZEB factors during hypoxia. However, the consequences of such a mechanism for both the development processes and the hypoxic cancer microenvironment will require further validation. 

Establishing the specific impact of hsa-miR-429 on ZEB factor expression is very challenging, since all miR-200 members are part of this negative feedback loop [138]. Furthermore, cancer-specific epigenetic changes such as silencing of Achaete-Scute Homolog (*ACLS2*) [96] or increasing the levels of lncRNA sponges such as MAPKAPK5 Antisense RNA 1 (*MAPKAPK5-AS1*) [94], circular RNA protein tyrosine kinase 2 (*circPTK2*) [115], *circLIFR* [116] or metastasis-associated lung adenocarcinoma transcript 1 (*MALAT1*) [99,117] that limit the miR-200 family expression, were also shown to correlate with an increased metastatic potential. The role of hsa-miR-429, however, in modulating EMT is not limited to the ZEB factors, since other direct targets include other important positive transcriptional regulators of this pathway such as *EZH2* [142], *RBBP4* [143] and *NOTCH1* [144]. Thus, depending on the cancer type, the reduction of hsa-miR-429 levels could also accelerate the EMT completely independently of the ZEB pathway. 

EZH2 has been shown to increase the expression of circRNA_0082835, an hsa-miR-429 sponge in cancer cells, creating another negative feedback loop-based regulation between EZH2 and hsa-miR-429 [123]. Interestingly, however, AKT1, that is a negative regulator of EMT [145], has been shown to be a direct target of hsa-miR-429, along with tight junction protein 1 (TJP1) and occludin (OCLN) that are repressed during the EMT process [146,147]. Thus, reduced hsa-miR-429 levels can lead to increased AKT1, TJP1 and OCLN levels and consequently limit the EMT, counteracting this miRNA effect on ZEB factors and other EMT-favoring targets.

Furthermore, HIF-1 acts as a master regulator of cellular adaptation to low oxygen levels and can also directly stimulate the expression of *ZEB1* [148,149,150] and modify Notch1 signaling [151,152,153]. Given that *HIF1A* forms a negative feedback loop with has-miR-429 [101], the interpretation of this miRNA role in the context of the hypoxic tumor microenvironment can be extremely challenging, given the HIF-1/ZEB1/Notch1 pathway crosstalk. Additionally, the expression of many lncRNAs that were proposed to reduce hsa-miR-429 levels, including *MALAT1, LINC01234, XIST, MAPKAPK5-AS1* and *PVT1,* and were shown to be altered during hypoxia should be considered [154,155,156,157,158,159,160,161]. 

Both hypoxia and the activation of HIF-1 signaling are common features of tumor microenvironments [162,163,164]. They lead to both hsa-miR-429 induction and the accumulation of lncRNAs that are capable of reducing this miRNA expression levels. Given the inhibitory effect of hsa-miR-429 on EMT, we speculate that HIF-1 tumor-specific impact on lncRNAs levels, e.g., *MALAT1* or *XIST*, could potentially help cancer cells to overcome the hsa-miR-429-related blockaid and shift the cells towards EMT. Furthermore, since oncogenic alterations in miRNA levels are often associated with drug resistance [165,166] and chemoresponses [167,168], the cancer-specific deregulation of HIF-1-related hsa-miR-429 induction may have a significant impact on therapeutic efficiency. This hypothesis, however, will require in-depth mechanistic tumor-specific studies in order to understand the impact of HIF-1-related tuning of hsa-miR-429 expression on the metastatic potential and the possibility of any viable drug responses. 

**Figure 4:**
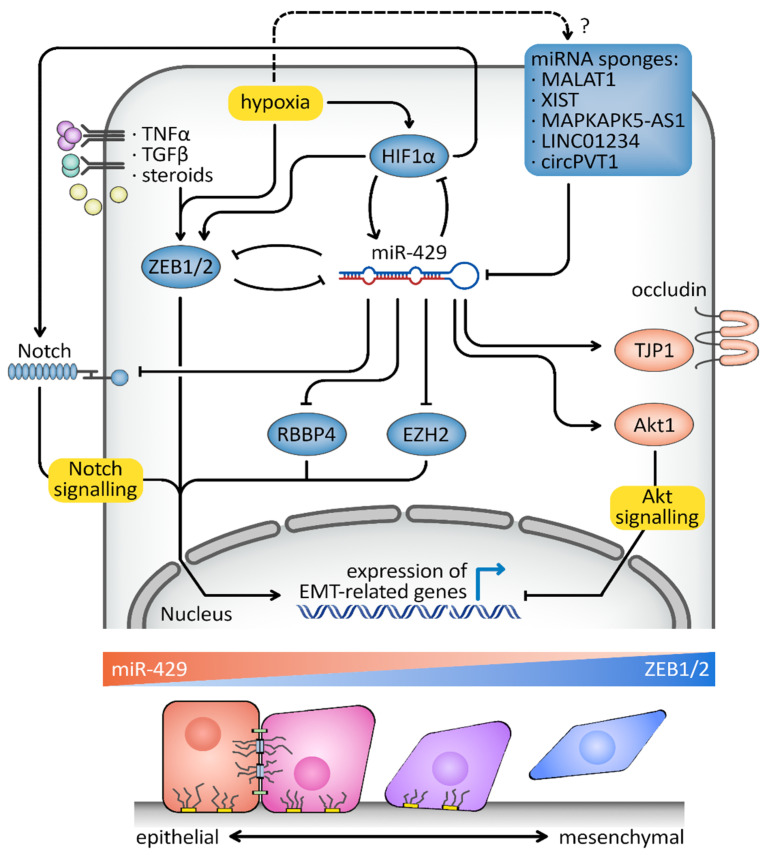
hsa-miR-429 is an important modular of EMT. hsa-miR-429 creates a negative feedback loop with ZEB1/ZEB2 transcription factors to reversibly direct cells to EMT. Furthermore, other hsa-miR-429 targets as well as transcriptional and posttranscriptional regulators of this miRNA expression modulate the extent of EMT signaling (see text for more details).

This complex contradictory mechanism of hsa-miR-429 involvement in EMT signaling (Figure 4) requires further study to determine the usefulness of miR-200 family members to modulate EMT-related responses. Nevertheless, considering that miRNA acts as a buffer in gene expression profiles, the complex interactions of hsa-miR-429 may be critical for the proper tuning of developmental processes that include neurogenesis. Indeed, *ASCL2*, which is an important regulator of cellular differentiation [169], has been shown to induce the expression of hsa-miR-429 [96]. Obviously, further research is necessary to determine if feedback loop-based changes in hsa-miR-429 levels can work as an EMT switch during development.

## 5. Responses to Hypoxia 

In both physiological and pathological conditions, the process of cellular adaptation to insufficient oxygen levels is based on the wide transcriptional reprogramming of gene expression. Reprograming is needed in order to alter metabolism toward glycolysis, prevent cell death and restore oxygen homeostasis through the activation of angiogenesis and erythropoiesis [170]. The master regulators of these cellular activities are hypoxia-inducible factors (HIFs) that during hypoxia form active heterodimers of alpha subunits with their oxygen-independent beta subunits [171,172,173]. The HIF-1 complex mediates the initial and very wide reprograming of gene expression during acute hypoxia [174]. Extensive cellular adaptation to low oxygen levels, along with the intensive effort to restore the normal oxygen pressure through the upregulation of angiogenesis and erythropoiesis [50,51,174], creates a potential risk of oxidative damage (reoxygenation injury) upon the rapid restoration of the cellular oxygen levels [175]. 

During chronic hypoxia in endothelial cells in vascularized organs such as the lung, heart, placenta and kidney [176,177,178], the other HIF isoform, HIF-2α (*EPAS1*), eventually replaces HIF-1 and modulates its activity to a more balanced adaptative response [173,179,180,181]. Importantly, passing the transcriptional signal from HIF-1 to HIF-2 is necessary for proper vascularization during development [182,183]. However, the switch from HIF-1 and HIF-2 is also utilized by many cancer cells as an adaptive mechanism to the chronic oxygen deficiency in the tumor microenvironment [179,184]. Furthermore, another tissue-specific HIF alpha isoform, HIF-3α-2, accumulates during prolonged hypoxia in order to either support erythropoietin production in hepatoma cells [185] or induce the mTOR pathway in endothelial cells and eventually to direct the cells toward apoptosis [186,187]. 

Thus, the switch from HIF-1 to HIF-2 and HIF-3 is necessary for the proper adjustment of cellular homeostasis towards survival (HIF-2) or apoptosis (HIF-3) during prolonged hypoxia. Importantly, during hypoxia, the levels of both HIF-1 and HIF-3 are dynamically modulated by hsa-miR-429. Interestingly, both *HIF3A* and hsa-miR-429 are induced by HIF-1 [101,103,188], while *HIF1A* and *HIF3A* are direct targets of hsa-miR-429 [101,103]. Notably, HIF-2 levels are independent of HIF-1, whereas the *EPAS1* transcript (HIF-2) seems to be less prone to miRNA-related degradation [83].

This relationship between the HIFs and hsa-miR-429 has two important functional implications in endothelial cells. First, HIF-1 creates a negative regulatory loop with hsa-miR-429 that is responsible for decreasing the *HIF1A* message during hypoxia as the *EPAS1* levels increase. Secondly, it prevents an extensive *HIF3A* accumulation during acute hypoxia that could potentially limit HIF-1 activity and induce cell death. In contrast, during prolonged hypoxia, it attenuates the continuous HIF-1 transcriptional activity through the dominant negative properties of HIF-3α [189] as well as promotes the initiation of apoptosis [186]. 

**Figure 5:**
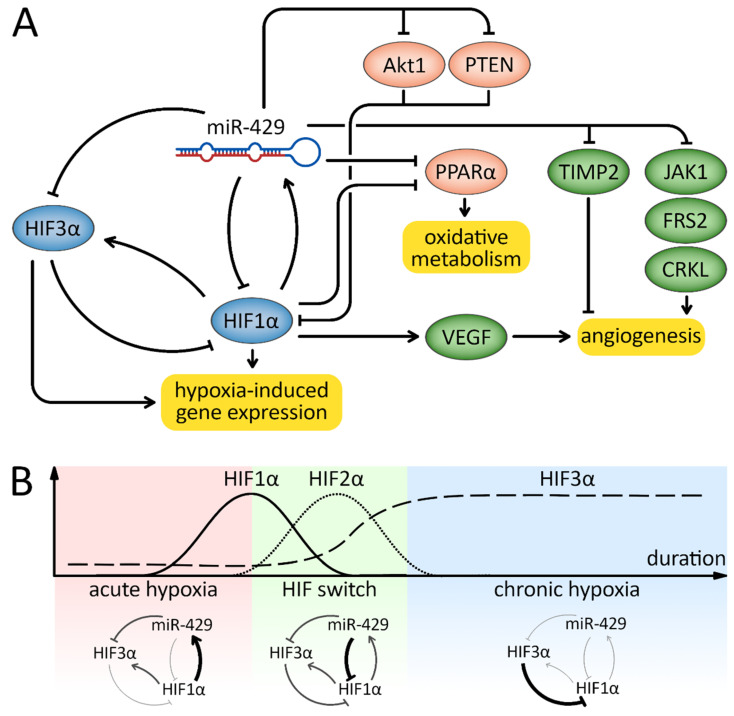
hsa-miR-429 is an important mediator of the adaptive response to hypoxia and angiogenesis. (**A**) hsa-miR-429 creates a negative feedback loop with HIF-1α transcription factor to tune the extent of HIF-1-mediated reprograming of gene expression in response to hypoxia. Furthermore, other hsa-miR-429 targets, as well as transcriptional and posttranscriptional regulators of this miRNA, tune the extent of the adaptive response to hypoxia, including angiogenesis (see text for more details). (**B**) hsa-miR-429 contributes to the switch from HIF-1 to HIF-2 and HIF-3. During acute hypoxia, the negative feedback loop between HIF-1 and miR-429 controls HIF-1 levels, prevents HIF-3 signaling and reduces *HIF1A* levels. This promotes the transition from HIF-1 to HIF-2. Furthermore, reduced levels of hsa-miR-429 during chronic hypoxia support HIF-3 accumulation (see text for more detail).

Taken together, in endothelial cells at least, hsa-miR-429 serves as a crucial modulator of HIF signaling. This miRNA maintains low levels of *HIF1A* and *HIF3A* expression during normal oxygen conditions, while during the early stages of hypoxia, when HIF-1α is dramatically elevated, hsa-miR-429 elevated expression maintains low levels of *HIF3A* mRNA and protein. During prolonged hypoxia, the HIF-1α levels decline via hsa-miR-429 mediated effects on *HIF1A* mRNA levels. This then leads to a reduction in has-miR-429 levels and a subsequent elevation of the *HIF3A* message and HIF-3α protein accumulation over time. Thus, hsa-miR-429 regulates the transitional switch between HIF-1-, HIF-2- and HIF-3-mediated transcriptional reprogramming in endothelial cells during hypoxia (Figure 5). Although the described mechanisms are well characterized in normal endothelial cells, it should be emphasized that they may also be active in the vessels of hypoxic solid tumors [190,191]. Therefore, the tumor microenvironment may be an important determinant of hsa-miR-429 pathological function. Importantly, additional hsa-miR-429 mRNA targets were reported in hypoxia-exposed cancer cells, stressing this miRNA role in controlling both HIF signaling and hypoxia-induced angiogenesis. Similar to what was found for EMT, AKT1 as an hsa-miR-429 target, was shown to limit HIF-1α expression [192]. Thus, the induction of hsa-miR-429 by HIF-1 during acute hypoxia can serve a similar role as that of HIF-3 and prevent AKT1-related HIF-1 inactivation early during hypoxia, while this miRNA’s decline during prolonged hypoxia could facilitate the HIF switch. 

Additional levels of hsa-miR-429 impact on the HIF pathway can also be provided by the direct modulation of PTEN levels [129]. PTEN is a repressor of HIF-1 transcriptional activity [193], and interestingly, the loss of expression of this gene is observed in many types of cancers [194]. Hence, hypoxic changes in hsa-miR-429 can also modulate the extent of HIF-1 transcriptional activity. Furthermore, the hypoxic elevation of hsa-miR-429 can also contribute to HIF-1-mediated reduction in peroxisome proliferator-activated receptor alpha (PPAR-α) expression during hypoxia [195]. This mechanism complements the role of the HIF switch in the adaptative response to hypoxia, since a decrease in PPARα will increase the efficiency of cellular oxygen use through the modulation of mitochondrial metabolism by decreasing the beta-oxidation of fatty acids [196]. 

hsa-miR-429 can also modulate the extent of hypoxia-induced angiogenesis indirectly by controlling the extent of HIF signaling and thus *VEGF* expression [197]. hsa-miR-429 can also modulate angiogenesis through direct interactions with other regulators including v-Crk avian sarcoma virus CT10 oncogene homolog-like (*CRKL*) [198], fibroblast growth factor receptor substrate 2 (*FRS2*) [199], tissue inhibitor of metalloproteinases 2 (*TIMP2*) [200] and Janus kinase 1 (*JAK1*) [201]. Since some of these hsa-miR-429 targets either have antiangiogenic functions (e.g., TIMP2) [200] or are cancer-type specific, further studies are necessary in order to understand how this miRNA modulates angiogenesis in different types of cancer. 

As already mentioned, numerous lncRNAs that sequester (sponges) hsa-miR-429, such as *MALAT1, LINC01234, XIST, MAPKAPK5-AS1* and *PVT1*, are dysregulated by hypoxia (usually, induced) [154,155,156,157,158,159,160,161] or promote oncological transformation [202]. However, many of them bind many other miRNAs under the very same conditions, including all miR-200 family members [203]. Our understanding of their dynamic changes during hypoxia and their preferences and efficiency in binding specific miRNAs in more physiologically relevant models remains limited. Thus, more studies are needed to establish how effective these lncRNAs are in terms of reducing the cellular hsa-miR-429 expression levels when other mRNA targets are available. 

## 6. Discussion

Taken together, hsa-miR-429 appears to be critical for the regulation of cellular adaptation to hypoxia and EMT. Both of these processes are extremely important for development and for maintaining cellular homeostasis. Since these aspects of cell biology are dysregulated in human pathologies such as cardiovascular disease and cancer, understanding the role of hsa-miR-429 could be a starting point for novel therapeutic and diagnostic strategies. Nevertheless, understanding the complex and overlapping networks of hsa-miR-429 mRNA target genes that are master transcriptional factors for EMT and HIF pathways remains a difficult task. Performing mechanistic studies that would involve all of them simultaneously is currently beyond the current research possibilities. 

Importantly, this problem can be partially solved by the proper identification of hsa-miR-429 direct targets and by analyzing the kinetics and priority of specific miRNA–mRNA interactions within signaling pathways, as well as the related changes in hsa-miR-429 levels and other miR-200 family members that share the same or very similar seed sequences. There are only a few reports, however, that utilize such an approach. Unfortunately, a plethora of proposed hsa-miR-429 targets were not rigorously verified and are often downstream targets of transcription factors that are themselves direct targets of hsa-miR-429. Furthermore, in terms of cancer cell lines, both miR-200 family members as well as the related signaling pathways can be affected by oncogenic transformation in a cancer/patient-specific manner, and generalizing the observation regarding hsa-miR-429 role requires direct verification in various cancer cell lines. 

Taken together, despite hsa-miR-429 role (along with that of the other miR-200 family members) as an important regulator of both physiological and pathological processes, more rigorous and extensive studies are needed to decipher the network of has-miR-429 molecular targets and to estimate this miRNA family’s role on cellular homeostasis. Furthermore, since both EMT and the cellular response to hypoxia are important aspects of development, hsa-miR-429 may also be involved in the modulation of these processes. Nevertheless, only a few studies have considered the role of this miRNA in development [152,204,205], and therefore more research is needed to verify this regulatory role.

## 7. Conclusions

Although the role of miRNAs in the regulation of both physiological and pathological aspects of cell biology is undisputable, translation of these ncRNAs into the clinic remains difficult. As discussed here with the hsa-miR-429 example, the main limitations of the development of efficient miRNAs-related therapies and diagnostic approaches are not only the complex networks involving these noncoding RNAs, but also the difficulties in identifying false positives. Thus, to provide miRNA-related clinical benefits, more rigorous and comprehensive research is needed to increase our understanding of the mechanisms underlying their biological role in models that are as physiologically relevant.

## Figures and Tables

**Table 1:** Experimentally verified hsa-miR-429 targets. (+) and (−) indicate the lack or presence of experimental approaches towards miRNA direct binding validation; (*) indicates an miRNA sponge. If Digital Object Identifier (DOI) was not available, the Pub Med ID (PMID) was used.

DOI	Targets	Sponge (*)	Transcriptional Regulator	Mimic	Inhibitor	Luciferase Reporter	Target Protector	RIP	qPCR	WB	Animal Model	miR-200 Family Verification	Reference
ttAV10.1038/ncb1722	*ZEB1* *ZEB2*	−	ZEB1	+	+	+	−	−	+	+	+	+	[91]
10.1007/s12013-014-9885-8	*ZEB1*	−	−	+	+	+	−	−	+	+	+	+	[92]
10.1080/21655979.2021.1923238	*ZEB1*	−	−	+	+	+	−	−	+	+	+	−	[93]
10.1186/s12860-022-00420-x	*ZEB1* MAPKAPK5-AS1 *	+	−	+	+	+	−	+	−	+	+	−	[94]
Q31016/j.biopha.2016.03.035	*ZEB1* *CRKL*	−	−	+	+	+	−	−	+	+	+	−	[95]
10.1074/jbc.M114.598383	*ZEB1* *ZEB2*	−	ASCL2	+	+	+	−	+	−	−	−	+	[96]
10.1016/j.biocel.2019.05.021	*ZEB1* XIST *	+	−	+	+	+	−	−	+	+	−	−	[97]
10.1155/2021/7974012	*ZEB1* LINC01303 *	+	−	+	+	+	−	+	+	+	+	−	[98]
10.1016/j.lfs.2020.118480	*ZEB1* MALAT1 *	+	−	+	+	+	−	+	+	+	−	−	[99]
10.1097/CAD.0000000000001295	*LRP4* RNF185-AS1 *	+	−	+	+	+	−	+	+	+	+	−	[100]
10.1096/fj.14-267054	*HIF1A*	−	HIF-1	+	+	−	+	−	+	+	−	+	[101]
10.1007/s10529-018-2604-6	*HIF1A*	−	−	+	+	+	−	−	+	+	−	−	[102]
10.1038/srep22775	*HIF3A*	−	HIF-1	+	+	−	+	−	+	+	−	+	[103]
10.3892/ol.2020.11766	*HOXA9*	−	−	+	+	+	−	−	+	+	+	−	[104]
10.1016/j.abb.2017.11.011	*RAB23*	−	−	+	+	+	−	−	+	+	+	−	[105]
10.3389/fnmol.2018.00035	*TJP1* *OCLN*	−	−	+	+	+	−	−	+	+	−	−	[106]
10.1016/j.bbrc.2017.06.181	*RohE*	−	−	+	+	+	−	−	+	+	+	−	[107]
10.3892/mco.2019.1940	*AKT1*	−	−	+	+	+	−	−	+	+	+	−	[108]
10.3892/or.2015.4039	*PAK6*	−	−	+	+	+	−	−	+	+	−	−	[109]
10.1016/j.urolonc.2015.03.016	*BMI1* *E2F3*	−	−	+	+	+	−	−	+	+	−	−	[110]
10.3892/ijmm.2016.2558	*NOTCH1*	−	−	+	+	+	−	−	+	+	−	−	[111]
10.1093/carcin/bgt089	*RAB18*	−	HBx	+	+	+	−	−	+	+	−	+	[112]
10.3389/fonc.2020.01067	*CD274*	−	−	+	+	+	−	−	+	+	−	−	[113]
10.1089/cbr.2020.3705	*FRS2* SNHG6 *	+	−	+	+	+	−	+	+	+	+	−	[114]
10.1186/s12943-018-0889-7	*TRIM33* circPTK2 *	+	−	+	+	+	−	+	+	+	−	+	[115]
10.1007/s10528-022-10285-6	*CELF2* circLIFR *	+	−	+	+	+	−	+	+	+	+	−	[116]
10.1002/jcp.27772	MALAT1 *	+	−	+	+	+	−	+	−	+	+	−	[117]
PMID: 34786067	*SYNJ1* LINC01234 *	+	−	+	+	+	−	+	+	+	+	−	[118]
10.1155/2022/1447207	*JAK1* MSC-AS1 *	+	−	+	+	+	−	+	+	+	−	−	[119]
10.3892/mmr.2021.12323	*FOXK1* circPVT1 *	+	−	+	+	+	−	−	+	+	−	−	[120]
10.2147/OTT.S277284	*SCAMP1*	+	−	+	+	+	−	−	+	+	+	−	[121]
10.1016/j.lfs.2020.117323	*TRIB2* circ_0084043 *	+	−	+	+	+	−	+	+	+	+	−	[122]
10.1080/21655979.2021.1953822	EZH2 circRNA_0082835 *	+	−	+	+	+	−	+	+	+	−	−	[123]
10.1186/s11658-020-0202-9	*IKKB*	−	−	+	+	+	−	−	+	+	+	−	[124]
10.3892/mmr.2021.12220	*IKKB*	−	−	+	+	+	−	−	+	+	−	−	[125]
10.1016/j.omtn.2021.01.026	*PPARA*	−	EZH2	+	+	+	−	−	+	+	+	−	[126]
10.7150/jca.21024	XIST *	+	−	+	+	+	−	−	+	+	+	−	[127]
10.1016/j.bbrc.2014.05.084	*PTEN* *RASSF8* *TIMP2*	−	−	+	+	+	−	−	+	+	−	−	[128]
10.1016/j.canlet.2015.04.023	*PTEN*	−	−	+	+	+	−	−	+	+	+	+	[129]
10.1016/j.biopha.2020.110215	*CRKL*	−	−	+	+	+	−	−	+	+	−	−	[130]
10.1038/s41598-018-20258-8	*CRKL*	−	−	+	+	+	−	+	−	+	−	−	[131]
10.1136/gutjnl-2013-305715	*RBBP4*	−	−	+	+	+	−	−	+	+	−	+	[132]

## Abbreviations

Ago2 (Argonaute 2: AKT1 AKT serine/threonine kinase 1), ASCL2 (Achaete-Scute Family BHLH Transcription Factor 2), BMI1 (BMI1 proto-oncogene, polycomb ring finger), CD274 (CD274 molecule), CELF2 (CUGBP Elav-like family member 2), circADAM9 (circular RNA ADAM9_013), circEZH2 (circular RNA EZH2_027), circLIFR (circular RNA LIFR_002), circPTK2 (circular RNA PTK2_063), circPVT1 (circular RNA PVT1), CRKL (CRK like proto-oncogene adaptor protein), DOI (digital object identifier), E2F3 (E2F transcription factor 3), EMT (epithelial–mesenchymal transition), EPAS1 (endothelial PAS domain protein 1) (hypoxia inducible factor 2)), EZH2 (enhancer of zeste 2 polycomb repressive complex 2 subunit), FDR (false discovery rate), FOXK1 (forkhead box K1), FRS2 (fibroblast growth factor receptor substrate 2), HIF (hypoxia-inducible factor), HIF1A (hypoxia-inducible factor 1 subunit alpha), HIF3A (hypoxia-inducible factor 3 subunit alpha), HOXA9 (homeobox A9), IKBKB (inhibitor of nuclear factor kappa B kinase subunit beta), JAK1 (Janus kinase 1), LINC01234 (long intergenic non-protein coding RNA 1234), LINC01303 (long intergenic non-protein coding RNA 1303), lncRNA (long non-coding RNA), LRP4 (LDL receptor-related protein 4), MALAT1 (metastasis-associated lung adenocarcinoma transcript 1), MAPKAPK5-AS1 (MAPKAPK5 antisense RNA 1), miR (microRNA), mirDIP (microRNA Data Integration Portal), miRNA (microRNA), MSC-AS1 (MSC antisense RNA 1), mTOR (mammalian target of rapamycin), NGS (next-generation sequencing), NOTCH1 (notch receptor 1), OCLN (occludin), PAK6 (p21 (RAC1) activated kinase 6), PPARA (peroxisome proliferator activated receptor alpha), premiR (pre-microRNA), pre-miRNA (pre-microRNA), pri-miRNA (primary microRNA), PTEN (phosphatase and tensin homolog), qPCR (quantitative (real-time) polymerase chain reaction), RAB18 (RAB18, member RAS oncogene family), RAB23 (RAB23, member RAS oncogene family), RASSF8 (Ras association domain family member 8), RBBP4 (RB binding protein 4, chromatin remodeling factor), RIP (RNA immunoprecipitation), RISC (RNA-induced silencing complex), RNAi (RNA interference), RND3 (Rho family GTPase 3), RNF185-AS1 (RNF185 antisense RNA 1), SCAMP1 (secretory carrier membrane protein 1), SNHG6 (small nucleolar RNA host gene 6), ssRNA (single-stranded RNA), SYNJ1 (synaptojanin 1), TF (transcription factor), TGF-β transforming growth factor beta, TIMP2 TIMP metallopeptidase inhibitor 2, TJP1 (tight junction protein 1), TNF-α (tumor necrosis factor alpha, TRIB2 (tribbles pseudokinase 2), TRIM33 (tripartite motif containing 33), UTR (untranslated region), VEGF (vascular endothelial growth factor), XIST (X inactive specific transcript), ZEB1 (zinc finger E-box binding homeobox 1), ZEB2 (zinc finger E-box binding homeobox 2).
